# Supplementary material for: Enhanced TfR1 Recognition of Myocardial Injury after Acute Myocardial Infarction with Cardiac Fibrosis via Pre-Degrading Excess Fibrotic Collagen
Source: Biology (Basel). 2024 Mar 25;13(4):213. doi: 10.3390/biology13040213 (PMC11048469; doi:10.3390/biology13040213)

Supplementary Materials for

Enhanced TfR1 recognition of myocardial injury after acute myocardial infarction with cardiac fibrosis via pre-degrading excess fibrotic collagen

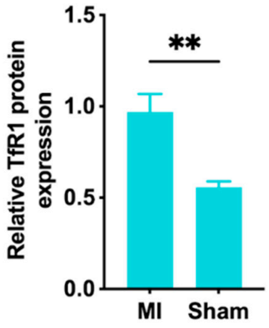

Figure S1. The western blot analysis of TfR1 in in MI and Sham group. (n=3, Student's *t* test. ** $p < 0.01$).

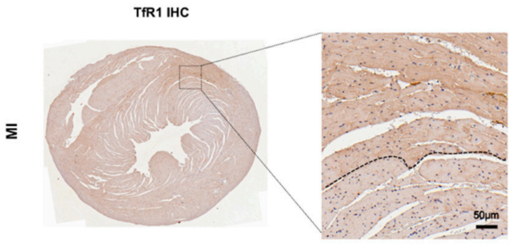

Figure S2. A high level of TfR1 expression was detected in the heart tissue of the MI mouse model by immunohistochemical staining. (Scale bar = 50 μm).

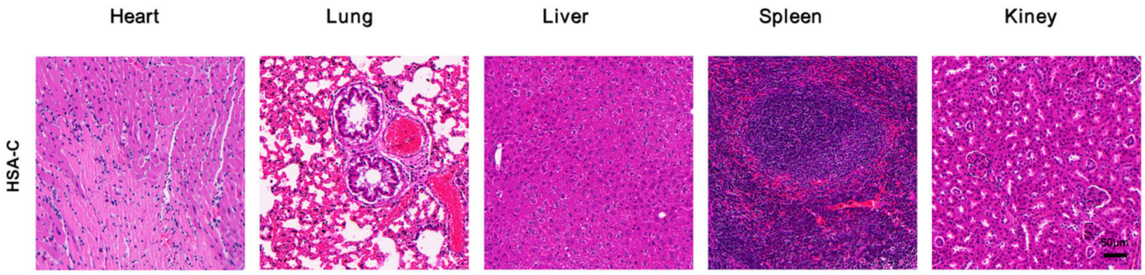

Figure S3. H&E staining of major organs in mouse which were pretreated with HSA-C. (Scale bar: 50 μm).

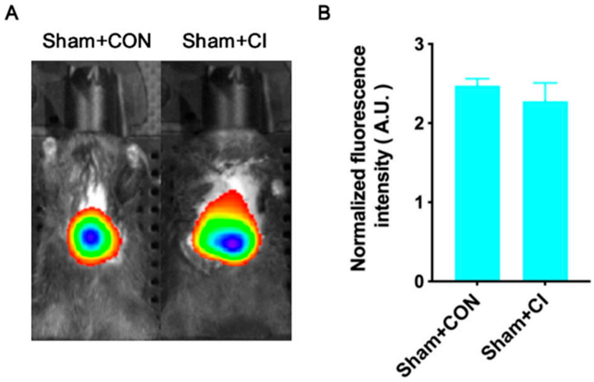

Figure S4. (A) The NIR fluorescence images of the MI mouse model at 48 h post-injection from different groups (Sham + CON *vs.* Sham + CI; n = 3). (B) Quantitative comparison of normalized fluorescence intensities of different group (Sham + CON *vs.* Sham + CI; n = 3).

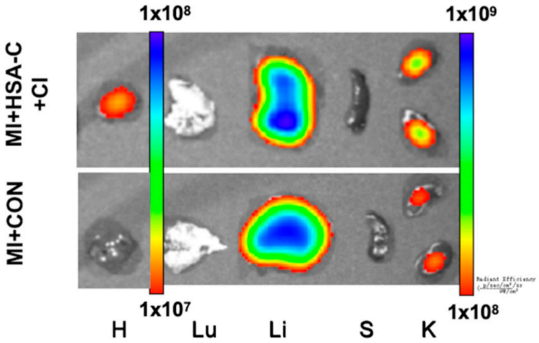

Figure S5. The *ex vivo* imaging for the heart and the other major organs of the MI mouse model (MI + CON *vs.* MI + HSA-C + CI), which were removed and captured 48 h after injection, were obtained using NIR fluorescence (H: Heart; Lu: Lung; Li: Liver; S: Spleen; K: Kidney).

Figure S6. Figure of Western Blot results: Comparison of TfR1 protein expression between myocardial infarction group and sham operation group.

TfR 1-GAPDH TfR1

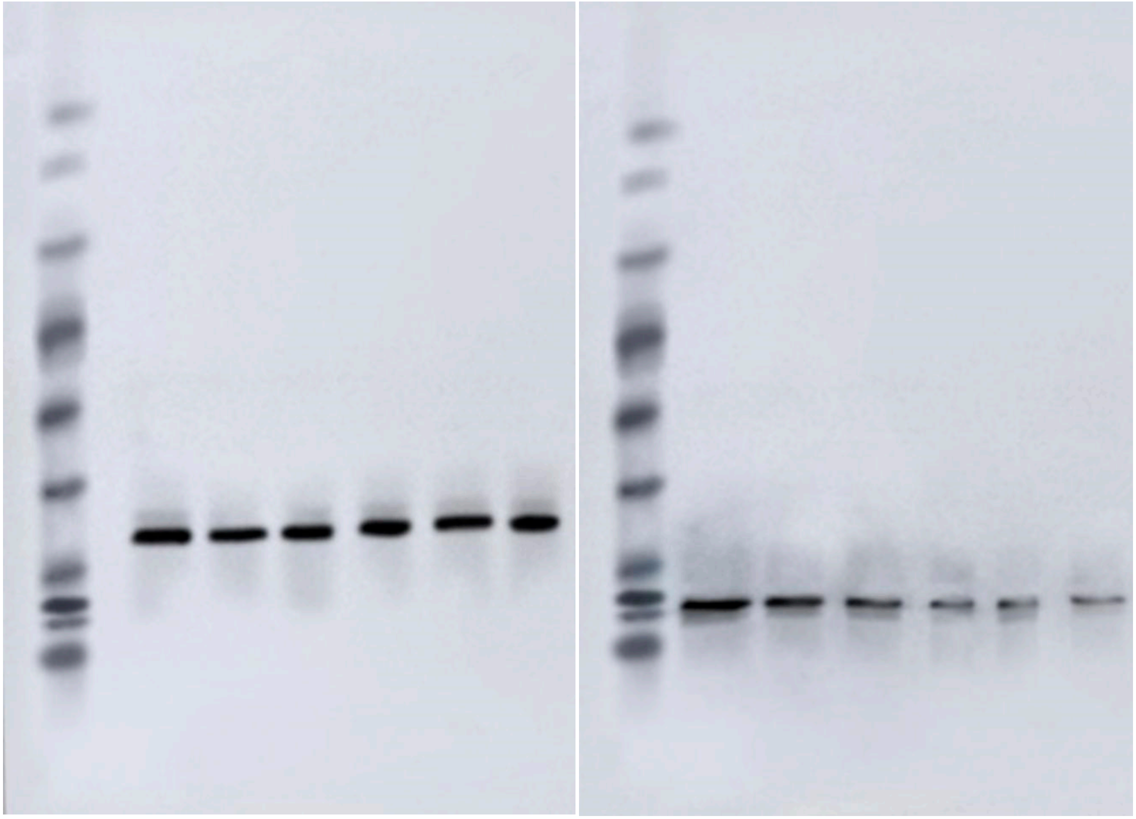

Supplement: Supplementary file 1 [file biology-13-00213-s001.zip › Supplementary Materials 0318.pdf]
